# Supplementary material for: Perforin and Granzyme B Expressed by Murine Myeloid-Derived Suppressor Cells: A Study on Their Role in Outgrowth of Cancer Cells
Source: Cancers (Basel). 2019 Jun 11;11(6):808. doi: 10.3390/cancers11060808 (PMC6627828; doi:10.3390/cancers11060808)
Supplement: Supplementary file 1 [file cancers-11-00808-s001.pdf]

cancers

Article

Perforin and Granzyme B Expressed by Murine Myeloid-Derived Suppressor Cells: A Study on Their Role in Outgrowth of Cancer Cells

Inès Dufait [1,2], Julian Pardo [3,4], David Escors [5,6], Yannick De Vlaeminck [2], Heng Jiang [1], Marleen Keyaerts [7,8], Mark De Ridder [1,†] and Karine Breckpot [2,†,*]

[1] Laboratory of Translational Radiation Oncology Physics and Supportive Care, Department of Radiotherapy, UZ Brussel, Vrije Universteit Brussel. Laarbeeklaan 101, 1090 Brussels, Belgium; ines.dufait@vub.be (I.D.); jiangheng1981@gmail.com (H.J.); mark.deridder@uzbrussel.be (M.R.)
[2] Laboratory of Molecular and Cellular Therapy, Department of Biomedical Sciences, Vrije Universiteit Brussel, Brussels. Laarbeeklaan 103, 1090 Brussels, Belgium; yannick.de.vlaeminck@vub.be (Y.V.); karine.breckpot@vub.be (K.B.)
[3] Fundación Instituto de Investigación Sanitaria de Aragón/ Universidad de Zaragoza, Centro de Investigación Biomédica de Aragón. Calle de San Juan Bosco, 13, 50009 Zaragoza, Spain; pardojim@unizar.es
[4] Fundación Aragon I+D (ARAID), Universidad de Zaragoza, Aragón. Calle de San Juan Bosco, 13, 50009 Zaragoza, Spain
[5] Immunomodulation group, Navarrabiomed-Biomedical Research Centre, IdISNA. C/irunlarrea 3 Complejo Hospitalario de Navarra. 31008 Pamplona, Navarra, Spain; davidescors@gmail.com
[6] Rayne Institute, Division of Infection and Immunity, University College London. 5 University St, London, WC1E 6JF, United Kingdom
[7] In Vivo Cellular and Molecular Imaging Laboratory, Vrije Universiteit Brussel. Laarbeeklaan 103, 1090 Brussels, Belgium; marleen.keyaerts@vub.be
[8] Nuclear Medicine Department, UZ Brussel. Laarbeeklaan 101, 1090 Brussels, Belgium
† These senior authors contributed equally
* Correspondence: karine.breckpot@vub.be; Tel.: +32-2477-4565

Received: 09 April 2019; Accepted: 6 June 2019; Published: date

Abstract: A wide-range of myeloid-derived suppressor cell (MDSC)-mediated immune suppressive functions has previously been described. Nevertheless, potential novel mechanisms by which MDSCs aid tumor progression are, in all likelihood, still unrecognized. Next to its well-known expression in natural killer cells and cytotoxic T lymphocytes (CTLs), granzyme B (GzmB) expression has been found in different cell types. In an MDSC culture model, we demonstrated perforin and GzmB expression. Furthermore, similar observations were made in MDSCs isolated from tumor-bearing mice. Even in MDSCs from humans, GzmB expression was demonstrated. Of note, B16F10 melanoma cells co-cultured with perforin/GzmB knock out mice (KO) MDSCs displayed a remarkable decrease in invasive potential. B16F10 melanoma cells co-injected with KO MDSCs, displayed a significant slower growth curve compared to tumor cells co-injected with wild type (WT) MDSCs. In vivo absence of perforin/GzmB in MDSCs resulted in a higher number of $CD8^+$ T-cells. Despite this change in favor of $CD8^+$ T-cell infiltration, we observed low interferon-γ (IFN-γ) and high programmed death-ligand 1 (PD-L1) expression, suggesting that other immunosuppressive mechanisms render these $CD8^+$ T-cells dysfunctional. Taken together, our results suggest that GzmB expression in MDSCs is another means to promote tumor growth and warrants further investigation to unravel the exact underlying mechanism.

Keywords: MDSC; CD8+ T-cell; perforin; granzyme B; cancer

a

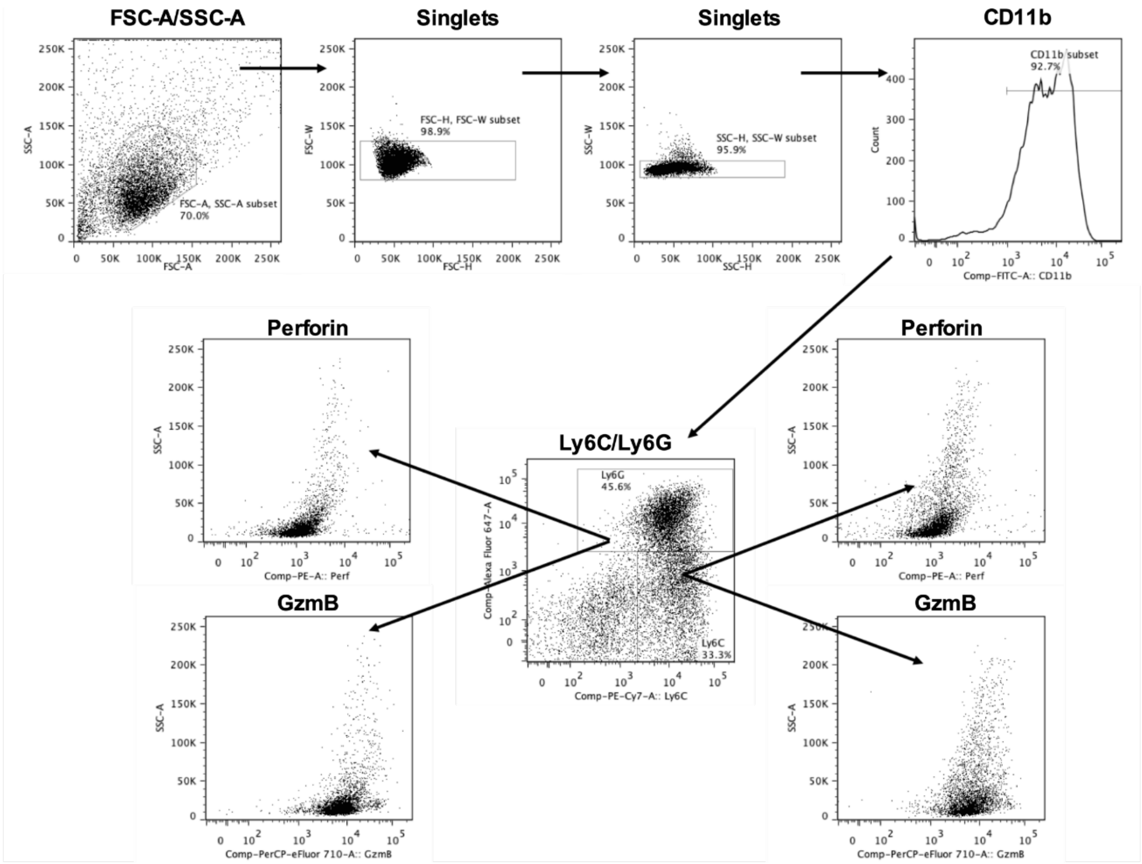

Supplementary Figure 1. In vitro myeloid-derived suppressor cells (MDSCs) express perforin and granzyme B (GzmB). MDSCs were generated starting from bone marrow of wild type (WT) mice using the conditioned medium (CM) of B16F10-GM-CSF cells. The gating strategy to delineate in vitro MDSCs (CD11b$^+$) versus monocytic (M)-MDSCs (CD11b$^+$Ly6C$^+$) and polymorphonuclear (PMN)-MDSCs (CD11b$^+$Ly6G$^+$) as well as the detection of perforin and GzmB is shown. The flow cytometry graphs are representative of at least three independent experiments.

© 2019 by the authors. Submitted for possible open access publication under the terms and conditions of the Creative Commons Attribution (CC BY) license (http://creativecommons.org/licenses/by/4.0/).
